# Postembryonic neuronal addition in Zebrafish dorsal root ganglia is regulated by Notch signaling

**DOI:** 10.1186/1749-8104-7-23

**Published:** 2012-06-27

**Authors:** Hillary Faye McGraw, Corey D Snelson, Andrew Prendergast, Arminda Suli, David W Raible

**Affiliations:** 1 Molecular and Cellular Biology Program, University of Washington, 1959 NE Pacific St, Seattle, WA, 98195, USA; 2 Neurobiology and Behavior Program, University of Washington, 1959 NE Pacific St, Seattle, WA, 98195, USA; 3 Department of Biological Structure, University of Washington, 1959 NE Pacific St, Seattle, WA, 98195, USA; 4 Department of Cell and Developmental Biology, Oregon Health and Science University, 3181 SW Sam Jackson Park Rd, Portland, OR, 97201, USA; 5 Department of Biology, University of Washington, 1959 NE Pacific St, Seattle, WA, 98195, USA

## Abstract

**Background:**

The sensory neurons and glia of the dorsal root ganglia (DRG) arise from neural crest cells in the developing vertebrate embryo. In mouse and chick, DRG formation is completed during embryogenesis. In contrast, zebrafish continue to add neurons and glia to the DRG into adulthood, long after neural crest migration is complete. The molecular and cellular regulation of late DRG growth in the zebrafish remains to be characterized.

**Results:**

In the present study, we use transgenic zebrafish lines to examine neuronal addition during postembryonic DRG growth. Neuronal addition is continuous over the period of larval development. Fate-mapping experiments support the hypothesis that new neurons are added from a population of resident, neural crest-derived progenitor cells. Conditional inhibition of Notch signaling was used to assess the role of this signaling pathway in neuronal addition. An increase in the number of DRG neurons is seen when Notch signaling is inhibited during both early and late larval development.

**Conclusions:**

Postembryonic growth of the zebrafish DRG comes about, in part, by addition of new neurons from a resident progenitor population, a process regulated by Notch signaling.

## Background

The neural crest, a transient population of vertebrate-specific progenitor cells, forms many disparate types of tissue after migration throughout the embryo (reviewed in [1,2]). In the trunk, neural crest gives rise to the pigment cells of the skin and the neurons and glia of the peripheral nervous system (PNS), including the sensory neurons and glia of the dorsal root ganglia (DRG). The DRG are located in register with somites along the lateral edges of the spinal cord, and convey sensory information to the dorsal horn. The DRG are established from a population of neural crest cells that migrate along a medial pathway between somites and neural tube, to form discrete ganglia.

Mammalian and avian DRG contain three types of sensory neurons: those that relay information about touch and limb position (mechanoreceptive and proprioceptive neurons) and those that convey information about painful and irritating stimuli (nociceptive neurons). In mammals and birds, development of the DRG occurs in successive waves (reviewed in [3-5]), regulated through the activity of two *neurogenin* genes [6]. The first wave depends upon the activity of *Neurogenin2* (*neurog2*), and gives rise to large-diameter mechanoreceptive and proprioceptive neurons. The second wave of neurogenesis is *Neurogenin1* (*neurog1*)-dependent and gives rise to mechanoreceptive and nociceptive neurons. A third wave of neurogenesis arises from migration and differentiation of *Krox20*-expressing boundary cap cells that differentiate into nociceptive neurons [7,8]. After neurogenesis is complete, cell death regulated by soluble neurotrophins refines the neuronal cell population to reach its final size before the end of embryogenesis.

Sensory neurogenesis in the zebrafish occurs in a somewhat different manner. Differentiation of neurons in the DRG is dependent on the activity of only one *Neurogenin* gene, *neurog1*[9], and no equivalent of *neurog2* exists in the zebrafish genome. DRG initially form with only two to five neurons differentiating from the neural crest by the end of embryogenesis. Adult animals have in the range of 100 neurons [10,11], and thus the vast majority of neurons must be added after embryogenesis. Zebrafish trigeminal sensory neurons born at different times generate different classes of neurons [12], suggesting that the postembryonic growth of the DRG might reflect a similar increase in complexity.

Here, we describe a mechanism by which zebrafish DRG neurons are added after embryogenesis. We find that neurons are added steadily throughout larval development in a process regulated by Notch signaling. In mammals, a subset of neural crest stem cells reside in an undifferentiated state in many adult tissues, including the DRG (reviewed in [13]), leading us to hypothesize that similar cells might be the source of additional neurons in zebrafish. Using *in vivo* lineage tracing, we find that some new neurons are generated from progenitor cells that are resident in the DRG.

## Results

### Neurons are continuously added to the larval dorsal root ganglia

**Figure 1:**
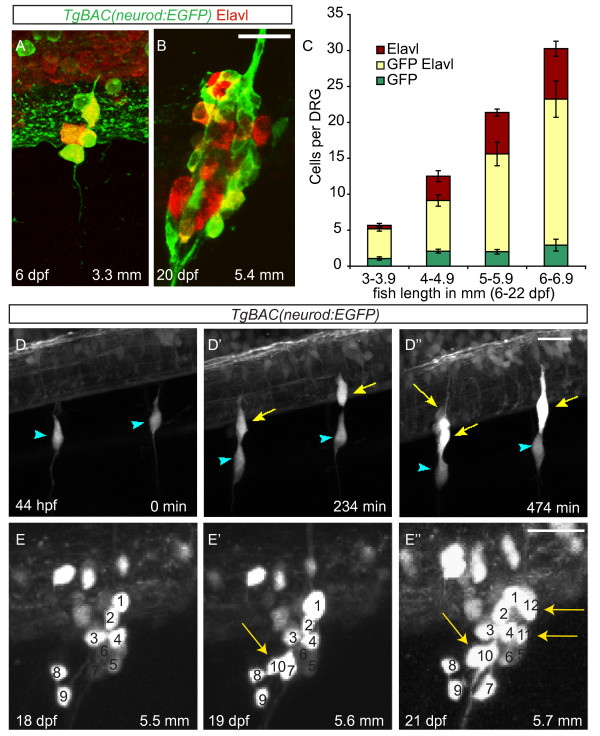
**Neurons are continuously added to the larval dorsal root ganglia (DRG). (A-B)** Confocal images of a *TgBAC(neurod:EGFP)* transgenic larva between six (**A**) and twenty-two dpf (**B**) showing DRG growth. Neurons were labeled with anti-Elavl (red) and GFP (green) antibodies. **(C)** Quantification (±SEM) of Elavl and GFP-positive cells per DRG in larvae staged by overall body length in millimeters (mm). Elavl-positive neurons are continuously added to the DRG as the larvae develop. Over half of the Elavl-labeled cells are also labeled with GFP (n = between five and fifteen larvae per condition, four ganglia per larva). **(-D”)** Stills from a time-lapse movie showing neuron differentiation in a *TgBAC(neurod:EGFP)* embryo between 44 and 52 hpf. (**D**) At 44 hpf the DRG contain a single neuron each (blue arrowheads). (**D’**) By 234 minutes, neurons have been added to each ganglion (yellow arrows). (**D”**) At 474 minutes the ganglia contain several neurons. **(E-E”)** Analysis of a single ganglion in a *TgBAC(neurod:EGFP)* transgenic larval fish between 18 and 21 dpf. (**E**) At eighteen dpf, the ganglion contains nine GFP-positive cells. (**E’**) At 19 dpf, the ganglion contains 10 GFP-positive cells (white arrow) and by 21 dpf (**E”**) 12 GFP-positive cells (white arrows). During the course of imaging the larval fish grew from 5.5 mm to 5.7 mm in length. Scale bars, 20 μm. dpf, days postfertilization; hpf, hours postfertilization; GFP, green fluorescent protein.

To determine whether neurons are added to the DRG in successive waves during larval development or if they are added steadily over time, we examined the total number of neuronal cells in the DRG during larval stages (six to twenty-two days postfertilization (dpf)). Larvae carrying the *TgBAC(neurod:EGFP)* transgene were stained with the pan-neuronal marker Elavl to label both nascent and fully differentiated DRG neurons (Figure 1A,B,C) and sorted by length in millimeters (mm) to more accurately compare fish at similar developmental stages [14]. At the earliest developmental stage examined (3.0 to 3.9 mm) there are an average of 5.7 ± 1.9 neurons per ganglion (n = 65 ganglia, 17 fish) (Figure 1C), and by the latest developmental stage examined (6 to 6.9 mm) there is an average of 30.5 ± 7.0 neurons per ganglion (n = 19 ganglia, 5 fish). The steady increase in the number of cells per ganglion demonstrates that neurons are continuously added to the DRG over larval stages (Figure 1C). To determine the pattern of neural addition during the initial formation of the DRG, we examined differentiation of sensory neurons using time-lapse imaging of the *TgBAC(neurod:EGFP)* line. Between 40 and 52 hours postfertilization (hpf), newly differentiating sensory neurons upregulate GFP, but do not divide (Figure 1D -D” Additional file 1). To look more closely at neuronal addition during larval development, live *TgBAC(neurod:EGFP)*-positive fish were examined over a period of four days when the fish were approximately 5.5 mm in length. In a representative fish, the ganglion initially contained nine neurons (Figure 1F), and during the duration of observation the ganglion added an additional four neurons (Figure 1F’,F”). Taken together, these results demonstrate that sensory neurons are added steadily to the DRG throughout the larval phase of development.

### New sensory neurons develop from progenitor cells within the dorsal root ganglia

**Figure 2:**
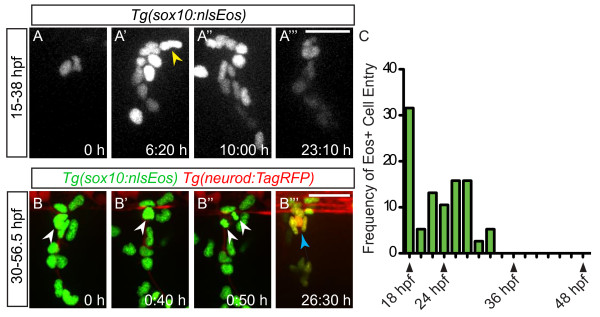
**Neural crest migration into the dorsal root ganglia (DRG) ceases prior to neuron differentiation. (A-A”’)** Confocal projections of stills from a time-lapse movie showing migrating *Tg(sox10:nlsEos)* + neural crest cells between 15 and 38 dpf. (**A**) At time zero, the region of DRG formation contains two Eos + cells. (**A’**) A still at 6:20 hours shows a representative neural crest cell joining the DRG anlagen (yellow arrowhead). Between 10:00 hours (**A”**) and 23:10 hours (**A”’**), no neural crest cells migrate into the DRG. **(B-B”’)** Time-lapse movie a *Tg(sox10:nlsEos)*/*Tg(neurod:TagRFP)* transgenic embryo between 30 and 57:10 hpf. (**B**) An Eos+/RFP- cell (white arrowhead) at time zero, begins to express RFP by 0:40 hours (**B’**) and divides at 0:50 hours (**B”**). (**B”’**) After 26.30 hours of imaging, neurons are labeled with RFP (blue arrow). **(C)** Quantification of Eos + cells that migrate into the DRG anlagen between 18 and 48 hpf. No new neural crest cells join the DRG after 32 hpf (n = five embryos). Scale bars, 25 μm. dpf, days postfertilization; hpf, hours postfertilization.

To determine which cells contribute to the expansion of sensory neurons, we analyzed the pattern of neural crest cell migration during nascent DRG formation. We used live imaging of cells in the *Tg(sox10:nlsEos)* transgenic line [15] to track the movement of neural crest cells. Between 18 and 32 hpf, *Tg(sox10:nlsEos)*-expressing neural crest cells migrate into the region of DRG formation (Figure 2 A-A”’,C; Additional file 2). Following the cessation of neural crest migration, a population of *Tg(sox10:nlsEos)*-positive cells remain in the DRG anlagen and form the neurons and glia of the DRG, and no additional cells are added through 56 hpf. Live imaging and cell tracking of a *Tg(sox10:nlsEos)/Tg(neurod:TagRFP)* embryo reveals that Eos+/RFP- cells divide and eventually give rise to RFP + DRG neurons (Figure 2B-B”’, Additional file 3). Together these results suggest that neural crest cells, as assayed by *Tg(sox10:nlsEos)* expression, end their migration into the DRG just prior to the onset of neurogenesis beginning at 36 hpf [9,16,17]. These results also demonstrate that the majority of neuronal addition occurs after neural crest migration ceases, and suggests that new neurons are derived from a population of cells within the DRG.

**Figure 3:**
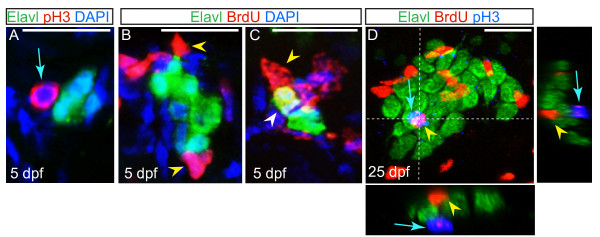
**Non-neuronal cells associated with the dorsal root ganglia (DRG) proliferate. (A)** Confocal projection of a ganglion at five dpf, showing sensory neurons labeled with Elavl (green) and an associated cell labeled with pH3 (red; blue arrow). Nuclei were labeled with DAPI (blue). **(B-C)** Ganglia of larvae at five dpf following BrdU incorporation (red). Neurons are labeled with Elavl (red) and nuclei are labeled with DAPI (blue). (**B**) Following a BrdU pulse at five dpf, cells associated with the DRG incorporate BrdU (yellow arrowhead), but neurons do not. (**C**) In a larva that was pulsed with BrdU at two dpf and chased to five dpf, a BrdU + neuron is located in the DRG (white arrowhead). **(D)** A ganglion in a 25 dpf, 6.25 mm larval fish exposed to a pulse of BrdU at 25 dpf, shows BrdU + cells (red; yellow arrowhead) and pH3 labeled cells (blue; blue arrow) are intermingled among Elavl + neurons (green), no neurons are labeled with BrdU or pH3. Dashed lines indicate levels of XZ and YX insets. Scale bars, 20 μm. BrdU, bromodeoxyuridine; DAPI, 4',6-diamidino-2-phenylindole; dpf, days postfertilization. pH3, phosphohistone H3.

Previous studies analyzing embryonic and larval development in the zebrafish DRG suggested that fully differentiated neurons are a source of newly added neurons as assayed by either bromodeoxyuridine (BrdU) incorporation or anti-phosphohistone H3 (pH3) labeling [10,18]. To address which cells divide within the DRG in more detail, larval fish were pulsed with the thymidine analog BrdU to mark cells in S-phase and/or labeled with anti-pH3 antibody to mark cells in M-phase, and labeling was carefully examined by confocal microscopy. In contrast to previous research, we did not find evidence of large-scale neuronal proliferation in the DRG. In five dpf larvae that were labeled with anti-pH3 antibody, only a small percentage of DRG had pH3+ cells in proximity to Elavl + neurons and no overlap in labeling was present (Figure 3A; 19 of 256 DRG (6%) had one or more associated pH3+ cells, n = 10 larvae). To further assess the role that proliferation plays during neuronal addition to the DRG, we preformed both acute and pulse-chase analyses of BrdU incorporation. In larvae that were incubated in BrdU immediately prior to fixation at five dpf, the vast majority of BrdU + cells associated with the DRG did not express Elavl, though a small subset of BrdU+/Elavl + cells was identified (2/204; Figure 3B; Table 1). Larvae that were exposed to a pulse of BrdU at two dpf and BrdU incorporation was analyzed at five dpf showed a small, but not significant increase in the number of BrdU+/Elavl + cells (Figure 3C; Table 1). Incorporation was also assessed in DRG of late-stage larval fish (25 dpf) two hours after BrdU injection. The DRG at 25 dpf contained both BrdU + cells (an average of 6.47 ± 0.54 per ganglion) and pH3+ cells (an average of 0.23 ± 0.08 per ganglion), but careful analysis of individual confocal slices revealed that in no cases were these cells also Elavl + (there were an average of 19.42 ± 1.28 Elavl + per ganglion; ± SEM; n = 15 fish) (Figure 3D; Additional file 4). These data suggest that non-neuronal cells associated with the DRG are proliferating and may act as precursor cells that give rise to new neurons as the DRG expands.

**Table 1:** Proliferation of cells associated with the dorsal root ganglia (DRG)

**treatment.**	**Average number of cells per DRG at five dpf**					
	**BrdU incorporation at two dpf**			**BrdU incorporation at five dpf**		
	**Elavl+**	**BrdU+**	**Elavl+/BrdU+**	**Elavl+**	**BrdU+**	**Elavl+/BrdU+**
**DMSO**	3.26 ± 0.27	2.11 ± 0.47	0.09 ± 0.05 (4/144)	3.74 ± 0.20	1.98 ± 0.33	0.04 ± 0.02 (2/204)
**DAPT**	3.86 ± 0.17^a^	2.67 ± 0.47	0.19 ± 0.05 (9/187)	4.32 ± 0.15^b^	1.69 ± 0.41	0.04 ± 0.04 (2/226)

Animals were incubated with BrdU for 1.5 hours at two dpf or five dpf. Both sets of larvae were incubated in DMSO or DAPT from two to five dpf. Animals were euthanized and fixed at five dpf for analysis. There was a significant increase in the total number of Elavl + neurons after DAPT treatment, but no change in proliferation. Numbers represent mean cell counts from nine to ten larvae per condition and four to six ganglia per larvae, ±SEM, ^a^*P* = 0.05, ^b^*P* = 0.03, Student’s *t* test. The number of BrdU+/Elav + compared to total Elav + cells is also shown in parentheses. BrdU, bromodeoxyuridine; DAPT, *N*-`*N*-(3,5-difluorophenacetyl)-L-alanyl]-*S*-phenylglycine t-butyl ester; DMSO, dimethyl sulfoxide; dpf, days postfertilization.

**Figure 4:**
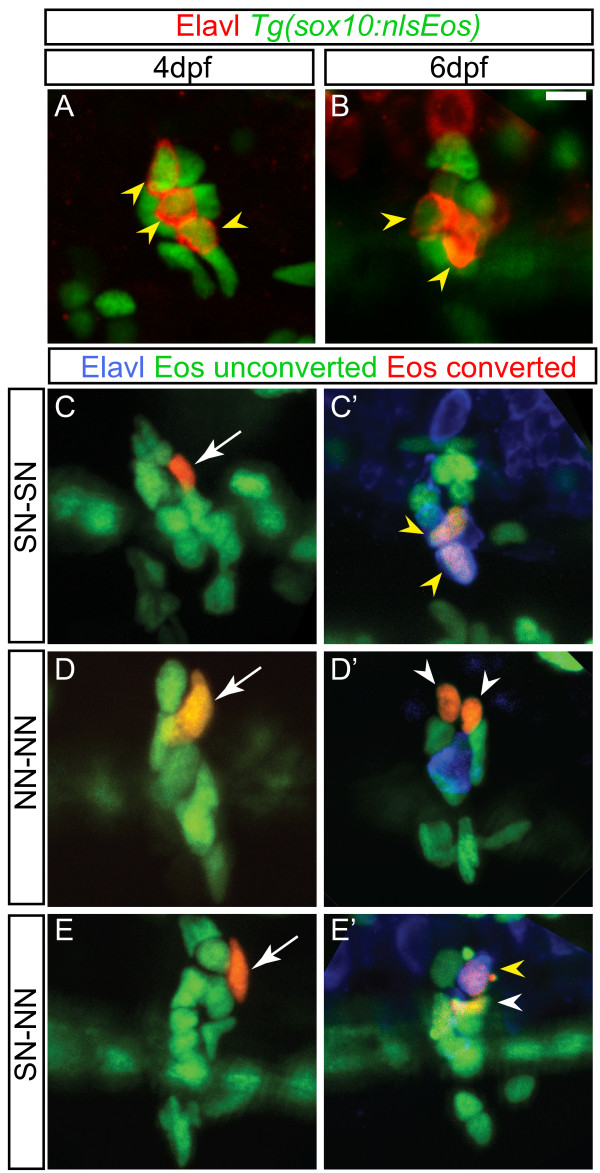
**Dividing progenitors within the larval dorsal root ganglia (DRG). (A-B)** Confocal projections of ganglia-containing cells that express the *Tg(sox10:nlsEos)* transgene (green) and neurons labeled with Elavl (red). At four dpf (**A**) and six dpf (**B**) *Tg(sox10:nlsEos)* labels both neurons (yellow arrowheads) and non-neuronal cells. **(C-E)***Tg(sox10:nlsEos)* cells were photoconverted by brief exposure to 405 nm light at four dpf, resulting in red fluorescence (white arrow). (**C’-E’**) At six dpf, larvae were labeled with Elavl (blue) and analyzed by confocal microscopy. Shown are examples of cells that divided and gave rise to two sensory neurons (SN-SN; yellow arrowheads) (**C-C’**), two non-neuronal cells (NN-NN; white arrowheads) (**D-D’**) or one sensory neuron and one non-neuronal cell (**E-E’**). Scale bar, 20 μm. dpf, days postfertilization.

To specifically address this hypothesis, we used the *Tg(sox10:nlsEos)* transgenic line to label and follow cells *in vivo*. The *sox10* promoter construct directs expression in migrating neural crest cells, in neural crest-derived glia and in cells fated to differentiate as sensory neurons [18]. The Eos protein undergoes irreversible photoconversion when exposed to 405 nm light [19], and the nuclear localization signal allows unambiguous labeling of single cells. Based on our findings that neural crest migration is complete prior to neurogenesis (Figure 2) and the findings of Carney *et al*. [18], we propose that the *Tg(sox10:nlsEos)* positive cells present in the DRG by four dpf includes non-neuronal cells comprised of satellite glial cells and progenitor cells; at present, there are no definitive zebrafish markers that allow us to distinguish between these cell types. Note also that the nls-Eos protein continues to be expressed in Elavl + cells, presumably because this protein is stable and persists, as Sox10 protein is downregulated in differentiated neurons [9]. At four dpf, the DRG contains an average six ± 0.19 *Tg(sox10:nlsEos)*-expressing cells and 2.8 ± 0.13 Elavl + cells (Figure 4A; ± SEM; n = five larvae). By six dpf, the DRG has expanded to contain an average eight ± 0.33 *Tg(sox10:nlsEos)*-expressing cells and 3.1 ± 0.16 Elavl + cells (Figure 4B; ± SEM; n = 13 larvae).

To identify the source of new neurons, we used photoconversion of Eos at four dpf to label cells associated with the DRG, and assessed their phenotypes at six dpf. Neurons and non-neuronal cells were distinguished by expression of Elavl. About two-thirds of labeled cells did not divide (Table 2). Of these, one-third was identified as sensory neurons, including some Eos + cells that were presumably already Elavl + at the time of photoconversion at four dpf. In some cases, photoconverted cells divided to generate two sensory neurons (Figure 4C,C’) or non-neuronal cells (Figure 4D,D’). Further, a few cells underwent asymmetric division to give rise to both sensory neurons and non-neuronal cells (Figure 4E,E’). These results demonstrate that some neurons are derived from dividing potential progenitor cells associated with the DRG.

**Table 2:** **Fates of labeled *Tg(sox10:nlsEos)* cells**

**CLONE TYPE**				
**SN**	**NN**	**SN-SN**	**NN-NN**^**a**^	**SN-NN**
23 (35%)	21 (32%)	4 (6%)	12 (18%)	6 (9%)

Cells in *Tg(sox10:nlsEos)* fish were photoconverted on four dpf and neuronal differentiation was assessed by immunolabeling with Elavl at six dpf. Only one cell was photoconverted per ganglion. Non-neuronal cells were defined as *Tg(sox10:nlsEos)* + cells that were Elavl-minus. SN, sensory neuron; NN, non-neuronal cell; SN-SN, two neurons; NN-NN, two non-neuronal cells; SN-NN, neuron + non-neuronal cell. ^a^Includes four clones that had three cells, suggesting cells underwent multiple divisions. Numbers in parentheses represent percentage of total.

### *delta* and *notch* are expressed in cells associated with dorsal root ganglia neurons

**Figure 5:**
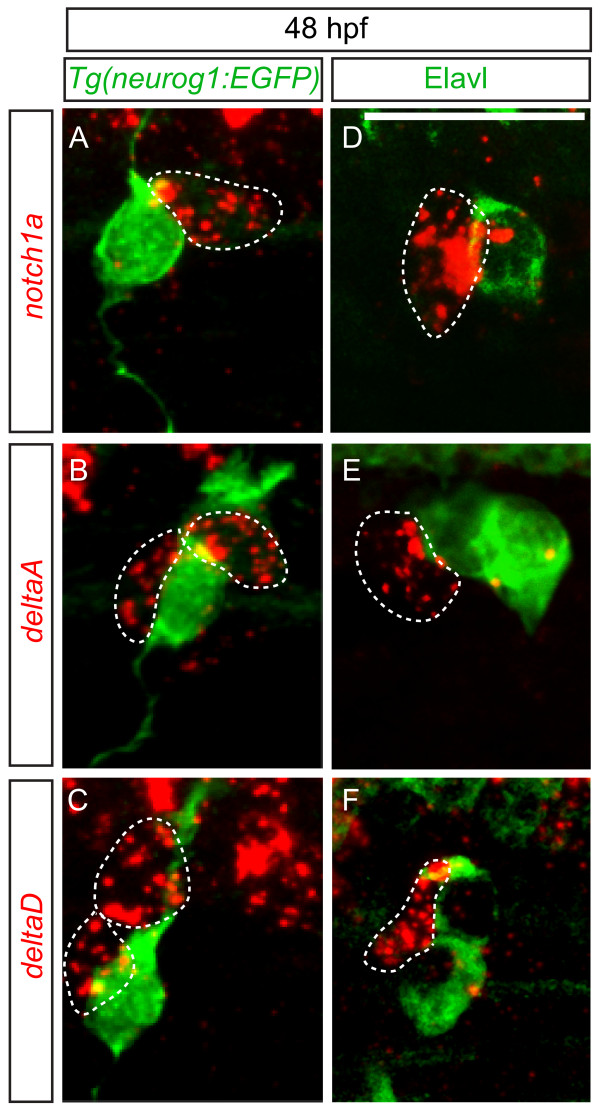
** *delta* and *notch* mRNAs are expressed in cells associated with dorsal root ganglia (DRG) neurons. (A-C)** Confocal images of *notch1a*, *deltaA* and *deltaD* fluorescent RNA *in situ* hybridization in 48 hpf *Tg(neurog1:EGFP)* transgenic embryos. (**A**) Expression of *notch1a* (red, dashed line) is found in a cell that neighbors GFP + DRG neuron (green). Both *deltaA* (**B**) and *deltaD* (**C**) are expressed in cells surrounding GFP + neurons. **(-F)** Comparison of anti-Elavl labeling and *notch1a*, *deltaA* and *deltaD* RNA expression at 48 hpf. (**D**) A cell expressing *notch1a* (red) is seen adjacent to an Elavl-positive neuron (green). (**E**) *deltaA* is expressed in cells surrounding Elavl-positive neurons, as is *deltaD* (**F**). Scale bars, 20 μm. hpf, hours postfertilization.

We next sought to uncover the mechanism underlying the differentiation of DRG neurons during larval development. The Delta/Notch signaling pathway is a central player in the establishment of neuronal fates in many systems (see [20,21] for review). Delta/Notch signaling has been shown to perform an important role in specification of neural crest and Rohon-Beard sensory neurons in the zebrafish [17,22], and specification of DRG neurons and glia in chick [23]. To determine whether the Delta/Notch signaling pathway may act to regulate the specification of zebrafish DRG sensory neurons from a progenitor population by a similar mechanism, we used fluorescent RNA *in situ* hybridization to identify the expression patterns of *notch1adelta*A and *deltaD* genes in the DRG at 48 hpf. We found that expression of *notch1adeltaA* and *deltaD* is seen in cells neighboring both *Tg(neurog1:EGFP)*-expressing cells (Figure 5A,-C) and Elavl + neurons (Figure 5D-F). These results suggest that non-neuronal cells associated with DRG might use the differential expression of *delta* and *notch* mRNAs to regulate progenitor cell differentiation.

### Inhibition of Notch signaling increases neuron number during larval dorsal root ganglia development

**Figure 6:**
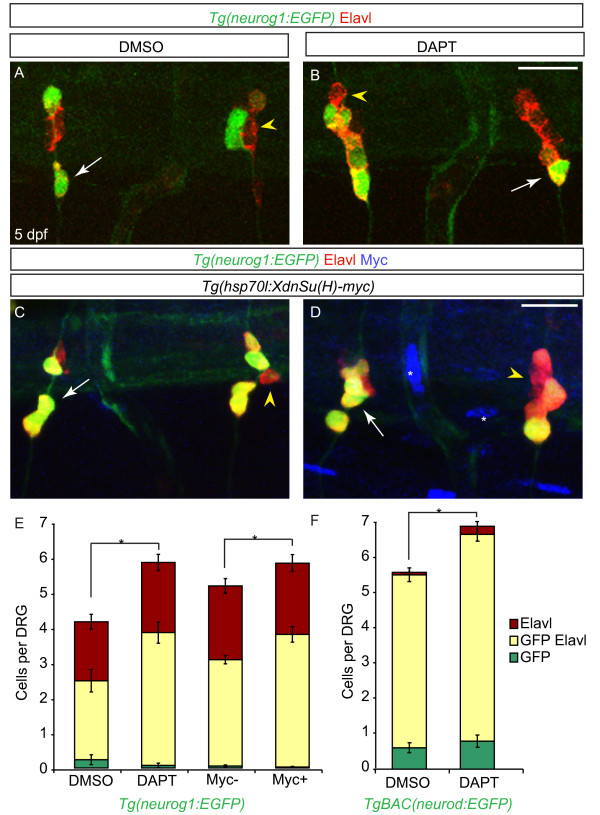
**Notch inhibition during larval development leads to an increase in dorsal root ganglia (DRG) neurons. (A-B)** Confocal projections of *Tg(neurog1:EGFP)* DRG neurons labeled with anti-GFP (green) and Elavl (red) from larvae treated with DMSO or DAPT between two and five dpf. At five dpf, DRG neurons are labeled by Elavl (yellow arrowhead) and a subset also express GFP (white arrow) in both DMSO (**A**) and DAPT (**B**) treated larvae. **(C-D)** Confocal images of *Tg(hsp70l:XdnSu(H)myc)*; *Tg(neurog1:EGFP)* larvae following heat-shock (HS) beginning at two dpf. In control (**C**) and transgenic (**D**) larvae at five dpf after HS, DRG neurons were labeled with GFP (green) and Elavl (red). Larvae carrying the *Tg(hsp70l:XdnSu(H)myc)* transgene were identified by ectopic myc labeling (blue, white asterisk) following HS. **(E)** Quantification of DRG neurons following Notch inhibition with DAPT or HS in *Tg(hsp70 DN (Su(H) myc)* embryos from two to five dpf. There is a significant increase in the number of DRG neurons in DAPT-treated and myc-positive larvae as compared to control larvae. (n = 6 for DMSO- or DAPT-treated larvae, n = 10 for myc-negative larvae and n = 13 for myc-positive larvae, five ganglia per larvae, *P <0.03, Student’s *t* test). **(F)** Quantification of DRG neurons in *TgBAC(neurod:EGFP)*-positive DRG following Notch inhibition with DAPT from two to five dpf. There is a significant increase in the number of DAPT-positive DRG neurons as compared to control embryos. (n = 9 for DMSO or n = 8 for DAPT-treated larvae, five ganglia per larvae, *P <0.005, Student’s *t* test). Scale bars, 20 μm. DAPT, *N*-[*N*-(3,5-difluorophenacetyl)-L-alanyl]-*S*-phenylglycine t-butyl ester; DMSO, dimethyl sulfoxide; dpf, days postfertilization GFP, green fluorescent protein.

Genetic disruption of Notch signaling results in loss of neural crest induction in developing zebrafish embryos [17,22], thus preventing the analysis of the requirement of this pathway at subsequent steps of development. To test the role of the Delta/Notch signaling pathway in specification of sensory neurons in the zebrafish DRG, we employed two methods of conditionally inhibiting Notch signaling. The gamma-secretase mediated cleavage of the Notch intracellular domain (ICD) was blocked with the pharmacological inhibitor DAPT, which has been shown to phenocopy Notch signaling mutants in zebrafish [24]. To confirm the efficacy of Notch inhibition by DAPT, we used transgenic line, *Tg(hsp70l:XdnSu(H)myc),* where a dominant-negative form of Suppressor of Hairless [25] is expressed following induction by heat-shock [26]. To determine whether the Delta/Notch signaling pathway acts after neural crest migration is complete, we continuously disrupted Notch signaling in *Tg(neurog1:EGFP)* larvae between two and five dpf. Treatment with DAPT during this period resulted in a significant increase in the number of DRG neurons as compared to DMSO-treated fish (Figure 6A,B,E). A similar result was seen using *Tg(hsp70l:XdnSu(H)myc)* larvae that were heat-shocked every 12 hours between two and five dpf (Figure 6C,D,E). Notch inhibition in a second transgenic line, *TgBAC(neurod:EGFP),* resulted in a comparable significant increase in the total number of GFP-positive and Elavl-positive neurons (Figure 6F).

To determine if proliferation is required for the increase in DRG neurons following Notch inhibition, we used acute or pulse-chase BrdU incorporation following the strategy described above. For the pulse-chase paradigm, larvae were treated with BrdU at two dpf and then incubated in DMSO or DAPT from two to five dpf. For acute BrdU experiments, larvae were exposed to DMSO or DAPT from two to five dpf and then treated with BrdU at five dpf just prior to fixation. Both DAPT treatment conditions showed a significant increase in the number of Elavl + neurons as compared to DMSO controls (Table 1). Following DAPT treatment, no significant increase was seen in BrdU + cells or BrdU+/Elavl + cells in either condition when compared to DMSO controls. These results suggest that blocking Notch signaling results in addition of DRG neurons primarily by promoting differentiation rather than increasing proliferation of progenitor cells.

### Addition of neurons in late-stage larval dorsal root ganglia is sensitive to Notch inhibition

**Figure 7:**
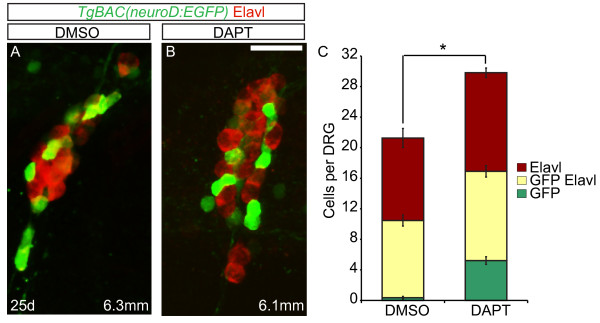
**Excess neurons differentiate in late larval dorsal root ganglia (DRG) following Notch inhibition. (A-B)** Confocal images of *TgBAC(neurod:EGFP)* transgenic fish treated with DMSO or DAPT between 20 and 25 dpf. DRG neurons were labeled with anti-GFP antibody (green) and Elavl (red). At 25 dpf, the DRG of a 6.3 mm DMSO-treated fish (**A**) and a 6.1 mm DAPT-treated fish (**B**) contain neurons that are labeled with Elavl, GFP/Elavl or GFP antibody alone. **(C)** Quantification of cells in the DRG. Following DAPT treatment, there is a significant increase in the total number of GFP + and Elavl + cells in the DRG as compared to controls. (n = 10 fish per condition, four ganglia per fish, *P <0.002, Student’s *t* test). Scale bars, 20 μm. DAPT, *N*-[*N*-(3,5-difluorophenacetyl)-L-alanyl]-*S*-phenylglycine t-butyl ester; DMSO, dimethyl sulfoxide; dpf, days postfertilization GFP, green fluorescent protein.

Zebrafish begin to transition from their larval-to-adult form during a metamorphic period that occurs between approximately 14 and 28 dpf based on changes in pigment pattern, physiology and behavior [14]. To determine whether the Delta/Notch signaling pathway continues to regulate neurogenesis during later stages, late larval fish were treated with DMSO or DAPT between 20 and 25 dpf. Both control and DAPT-treated larvae developed to an average of 6 mm in length. Despite being of similar developmental stage, the DRG of DAPT-treated fish contained significantly more neurons then DMSO-treated controls (an average of 29.8 and 21.3 respectively; n = 10 larvae per treatment group; *P* = 0.002; Figure 7A-C). Therefore, Notch signaling plays a role throughout larval development to regulate the specification of DRG neurons.

## Discussion

Zebrafish, like other aquatic vertebrates, undergo substantial growth after embryogenesis is complete, including in the size and complexity of their nervous system. We demonstrate the addition of new neurons to the zebrafish DRG is continuous during postembryonic (larval) growth. Similar increases in DRG neuron number have been reported during amphibian metamorphosis [27]. By contrast, growth of DRG in avian and mammalian species occurs during embryogenesis in overlapping waves that produce neurons of distinct function [6,28-32]. It remains to be determined whether distinct neuronal classes are added to the zebrafish DRG at specific developmental stages. While postembryonic growth of DRG in mammals has been controversial, addition of neurons in other mammalian PNS structures has been well documented [33,34].

Our lineage tracing experiments demonstrate that progenitors that reside among the Sox10-positive non-neuronal cells of the DRG are one source of new neurons during postembryonic growth. These results are consistent with a recent study demonstrating the addition of new DRG neurons from peripherally located precursors during avian embryogenesis [30]. In zebrafish, this process appears to continue long after embryogenesis has ended. We demonstrate that zebrafish progenitor cells are associated with the zebrafish DRG after neural crest migration is complete, although these cells share Sox10 expression with the neural crest cells from which they are derived. Previous studies in mammals have suggested continued addition of neurons during late embryonic development from other sources, including neural crest-derived boundary cap cells found at the dorsal and ventral roots [35-37]. Boundary cap cells can form a number of late-developing sensory neurons [7,8] and also acquire glial characteristics [38,39]. We believe that the progenitors we have identified in zebrafish are distinct from boundary cap cells, as they are located in positions distinct from the dorsal and ventral roots, and do not express *Krox20/egr2* mRNA (data not shown).

While sensory neurons are generally thought to be postmitotic, two previous studies have suggested that zebrafish DRG Elav + cells divide to give rise to new neurons [10,18]. These results are in contrast to our findings and to those in other systems [30]. We performed careful three-dimensional analysis to distinguish labeled cells, and note that while we have identified a very small number of Elav + cells that have incorporated BrdU, we do not find cells that are labeled with pH3. Furthermore, by time-lapse analysis we find that differentiated neurons do not divide, but rather arise de novo. We conclude that dividing Elav + cells are not a major source of DRG neurons in zebrafish.

We have not resolved whether DRG sensory neuron progenitors are bona fide glial cells, or whether they are a distinct population. Nervous system stem cells are now well established to have glial cell characteristics (reviewed in [40,41]), including those found in the PNS. We identified potential DRG progenitors by continued expression of the *Tg(sox10:nlsEos)* transgene and their positions adjacent to DRG neurons. Whether these cells acquire characteristics of satellite glia, which surround sensory neuron cell bodies and alter neuronal function [42,43], will require further study.

Our data suggests that Notch signaling regulates sensory neuron addition. We found that non-neuronal cells associated with differentiated DRG neurons express the Notch ligands *deltaA* and *deltaD*, as well as the Notch receptor *notch1a*. Inhibition of Notch signaling induces differentiation of DRG neurons in early- and late-stage larval fish. Our results are consistent with previous reports of an increase in DRG neuron number in zebrafish *notch1a* mutants [44]. Recent reports have also demonstrated that loss of Notch signaling increases the initial differentiation of DRG neurons from neural crest [45,46]. Our results suggest that Notch signaling normally prevents differentiation of neuronal progenitors. The expression of both ligand and receptor in subsets of cells adjacent to DRG neurons suggests a model where progenitor cells may act as an equivalence group: as cells begin to differentiate they inhibit their neighbors from doing so. One test of equivalence, whether cells are replaced by their neighbors after genetic or mechanical ablation, awaits future study. The methods we have used to block Notch signaling will act ubiquitously throughout the embryo, leaving open the possibility that effects are nonautonomous. In this scenario, blocking Notch would affect another cell type that would subsequently influence DRG development. Further experiments are needed to determine if Notch signaling acts cell autonomously within DRG progenitors.

Multiple roles for Notch signaling in neural crest development and differentiation have been previously identified, suggesting that this signaling pathway functions in a context-dependent manner. Notch signaling plays a role in the establishment of neural crest [17,22,47], and in the development of neural crest-derived cartilage and heart tissue [48]. Multiple roles for Notch signaling have also been described for glial cell specification and differentiation [49-52]. Here we describe a later role for Notch in regulating the differentiation of neuronal progenitors during postembryonic phases of zebrafish DRG development. Similar roles have been previously described for Notch signaling in avian sensory and sympathetic progenitors [53] and mouse enteric progenitors [54]. While the different roles for Notch signaling might be due to context or timing, the relative level of pathway stimulation may also have an effect [55]. In addition, oscillating expression of Notch signaling components alters the effects of this signaling pathway in the embryonic brain [56]. In cultures of neural crest stem cells, reduced levels of Notch signaling inhibit neurogenesis but do not promote gliogenesis, thus promoting self-renewal and maintaining pluripotency [57].

The presence of latent progenitors for postembryonic DRG growth is intriguing, and suggests a possible source of cells for regeneration. In mammals, neural crest-derived resident stem cells have been documented in a number of adult tissues, including amongst the enteric nervous system, heart, peripheral nerves and skin [58-64]. Neural crest stem or progenitor cells have also been isolated from embryonic and adult mammalian DRG [61,65-70]. These cells may be involved in replacement or repair instead of growth, the possible evolutionary origin of their function.

## Conclusions

Our work demonstrates the orderly addition of sensory neurons to the zebrafish DRG in the weeks after embryogenesis is complete, long after neural crest migration is complete. Neurons arise from dividing latent progenitor cells associated with the DRG that are amongst the satellite glial population. Progenitor differentiation is regulated by Notch signaling, throughout the period of larval development. The work provides insight into sensory neurogenesis in animals that undergo dramatic postembryonic growth.

## Methods

### Transgenic zebrafish lines

Larval and adult zebrafish were maintained at 28.5°C in a 14 hour/10 hour light–dark cycle with twice daily feedings. Embryos were generated from natural crosses between adults and staged in hours post fertilization (hpf) as described by [71]. Larval fish were staged in days postfertilization (dpf) and standard length [14]. The transgenic lines used were: *Tg(neurog1:EGFP)*^*w61*^ described in [9]; *TgBAC(neurod:EGFP)*^*nl1*^ described in [72], *Tg(sox10:nlsEos)*^*w18*^ described in [15] and *Tg(hsp70:XdnSu(H)myc)*^*vu21*^ described in [26]. Construction of the *Tg(neurod:TagRFP)*^*w69*^ transgenic line is described below. All work was approved by the University of Washington Institutional Animal Use and Care Committee.

### Construction of *Tg(neurod:TagRFP)*

The *Tg(neurod:TagRFP)*^*w69*^ transgenic line was constructed using the Multisite Gateway system [73]. The p5E-neuroD construct was generated using a 5 kb region of the *neurod* 5′ promoter ([72]; a gift from Teresa Nicolson). The p5E-neuroD construct was recombined with pME-TagRFP (a gift from Chi-Bin Chien) and p3E polyA. The construct was injected into one-cell embryos with Tol2 polymerase [74]. Injected embryos were raised to sexual maturity and screened by pair-wise crosses to obtain germ line transgenic fish that generated the expected expression pattern. The final construct was injected into one-cell zygotes, which were then raised to maturity and screened for progeny that carried the *Tg(neurod:TagRFP)* transgene in DRG neurons amongst other cells.

### Whole-mount immunohistochemistry and RNA *in situ* hybridization

For immunohistochemistry, larvae were collected at the stages indicated, euthanized in MS-222 (Sigma-Aldrich, St Louis, MO, USA); 10 mg/ml in buffered embryo medium) and fixed in 4% paraformaldehyde (PFA) in phosphate buffered saline (PBS) for two hours at room temperature. Antibody labeling was carried out as previously described [75]. In brief, embryos were washed in PBS with 0.1% TritonX-100 (PBT), and blocked with the addition of 2% goat serum. Prior to blocking, fish were permeablized with three one-hour water washes. Fish were incubated in primary antibodies diluted in blocking solution overnight at room temperature (RT). Primary antibodies used were anti-GFP (1:700; rabbit or mouse anti-GFP; Invitrogen, Carlsbad, CA, USA), anti-Elavl (1:700; monoclonal antibody (mAB) 16A11; also called anti-HuC/D; [76]; Invitrogen); anti-myc tag mAB (1:500, Cell Signaling Technology, Beverly, MA, USA), anti-5-bromo-2-deoxyuridine (rat anti-BrdU; 1:100; Abcam, Cambridge, UK) and anti-phosphohistone H3 (rabbit anti-pH3; 1:100, Cell Signaling Technology). Fish were incubated in Alexa-488, Alexa-568 or Alexa-647 conjugated secondary antibodies (Invitrogen) overnight at room temperature, rinsed in PBT and then stored in 50% glycerol/PBS for imaging. Nuclei were visualized by DAPI labeling (Invitrogen).

Fluorescent RNA *in situ* hybridization was performed as described [77]. Fixed larvae were made permeable for 20 minutes at RT using 10 μg/ml Proteinase K (Sigma-Aldrich) in PBT and then refixed for 15 minutes in 4% PFA. All hybridizations were carried out at 55°C. Digoxygenin-labeled antisense probes were generated using the following restriction enzyme and polymerases: *XbaI*/T7 for *notch1a*[78]; *EcoR1/T7* for *deltaA* and *deltaD*[79,80]. Following *in situ* hybridization, larvae were processed for immunohistochemistry as described above. Larvae were then stored in 50% glycerol/PBS for imaging.

### Inhibition of Notch signaling

The pharmacological inhibitor of gamma-secretase activity *N*-*N*-(3,5-difluorophenacetyl)-L-alanyl]-*S*-phenylglycine t-butyl ester (DAPT; [24]; Sigma-Aldrich) was used to conditionally inhibit Notch signaling. DAPT was dissolved in dimethylsulfoxide (DMSO) at 10 mM and a working stock was diluted to 100 μM in embryo medium (EM) as previously described [81]. Control fish were mock-treated with 1% DMSO in EM, for the same time course as the DAPT-treated fish. Zebrafish larvae were examined for the role of Notch signaling at two time points, either during early larval development, between two and five dpf, or during late larval development, between twenty and twenty-five dpf. Fish were treated continuously with DAPT or DMSO and were fed daily for the duration of the treatment. Following fixation, fish were processed for immunohistochemistry.

To corroborate the results seen with DAPT treatment, a heat-shock inducible dominant negative Suppressor of Hairless transgenic, *Tg(hsp70l:XdnSu(H)myc)*, was used [26]. Larvae were heat-shocked at 40°C for one hour twice daily between two and five dpf, fixed in 4% PFA, and processed for immunohistochemistry. Activation of the transgene was confirmed by the presence of myc labeling with an anti-myc antibody (Invitrogen).

### Cell cycle analysis

To assess cell cycle in the DRG, we used 5-bromo-2-deoxyuridine (BrdU) incorporation to label cells in S-phase and anti-phosphohistone H3 (pH3) antibody to label cells in M-phase [82]. In larvae, BrdU incorporation was carried out using a modified established protocol [83] Larvae were incubated in 10 mM BrdU dissolved in EM and 10% DMSO for 30 minutes at 4°C followed by one hour at 28.5°C. For pulse-chase experiments, larvae were exposed to BrdU between 50 and 51.5 hpf, transferred to DAPT or DMSO until five dpf and then fixed and processed. For pulse experiments, larvae that had been treated with DAPT or DMSO between two and five dpf, were exposed to BrdU at five dpf using the above protocol and then fixed and processed for BrdU detection. For BrdU incorporation in older larval fish, animals at 25 dpf were anesthetized in MS-222 and injected intraperitoneally using a glass capillary needle with 10 nl of a 10 mM BrdU in buffered Hank’s solution (protocol modified from [84]), incubated in EM at 28.5°C for two hours and then fixed for processing. BrdU detection was carried out using a previously describe protocol [85] Following BrdU detection, fish were immunolabeled with anti-Elavl and anti-pH3 antibodies and stored in 50% glycerol/PBS for imaging.

### *In vivo* fate mapping

Homozygous larvae carrying the *Tg*(*sox10:nlsEos)* transgene were raised to four dpf, anesthetized with MS-222, and mounted in 0.8% agarose on a glass-bottomed cell culture dish. Cells surrounding the DRG were exposed to 405 nm light on an Olympus FV1000 confocal microscope (Olympus, Tokyo, Japan) to convert individual green nuclei to red. Following photoconversion, larvae were released from the agarose, and incubated in either 100 uM DAPT or DMSO as a control. At six dpf, larvae were fixed in 4% paraformaldehyde for two hours at room temperature and labeled with anti-ElavI antibody, mounted in Vectashield (Vector Laboratories, Burlingame, CA, USA) and imaged.

### Data collection and time-lapse imaging

Prior to imaging, fish were mounted on bridged cover slips in 50% glycerol/PBS. Images and cell counts were obtained using a Zeiss LSM-5.0 Pascal confocal microscope (Carl Zeiss AG., Oberkochen, Germany), a Zeiss Axioplan 2 microscope and a Spot CCD camera (Diagnostic Instruments, Palo Alto, CA, USA), an Olympus FV1000 confocal microscope, or a 3I Marianas spinning disk microscope (Intelligent Imaging Innovations, Inc., Denver, CO, USA). Whole images were processed using ImageJ software (rsbweb.nih.gov/ij/index.html) and adjusted for brightness and contrast using Adobe Photoshop. For most individual cell counts, the five rostral-most DRG were analyzed in each condition. Non-neuronal cells were defined as associated with the DRG if they were in direct contact with labeled DRG neuronal cell bodies. Statistical significance was determined using Microsoft Excel software (Microsoft, Seattle, WA, USA) or GraphPad Prism version 5.0d software (Graphpad Software, San Diego, CA, USA).

For live imaging of neural crest migration and initial differentiation of neurons, embryos were anesthetized in MS-222, then embedded in 1.2% low melt agarose (Sigma-Aldrich) in EM. Cells were imaged every 10 minutes with a 20x water lens. In some cases stacks were assembled using Slidebook. Cells were tracked manually using ImageJ software. To image neuronal addition in larval fish, animal were anesthetized and embedded in 1.5% low-melt agarose and imaged using a 40x dipping lens. Following imaging, fish were released from the agarose and revived. Images were collected daily over a four-day time course.

## Abbreviations

BrdU, Bromodeoxyuridine; DAPI 4′,6-diamidino-2-phenylindole; DAPT*N*-[*N*-(3,5-difluorophenacetyl)-L-alanyl]-*S*-phenylglycine t-butyl ester; DMSO, Dimethyl sulfoxide; DRG, Dorsal root ganglion; dpf, Days postfertilization; EGFP, Enhanced green fluorescent protein; EM, Embryo medium; GFP ,Green fluorescent protein; hpf,Hours postfertilization; IVD, Intracellular domain; PBS, Phosphate buffered saline; PFA, Paraformaldehyde; pH3, Phosphohistone H3; RT, Room temperature; TagRFP, Tag red fluorescent protein.

## Competing interests

The authors declare that they have no competing interests.

## Authors’ contributions

HFM, CDS and DWR designed and analyzed experiments. HFM, AP and CDS performed experiments. AP, AS and AN developed key reagents. HFM, CDS and DWR wrote the manuscript. All authors read and approved the final manuscript.

## Supplementary Material

Additional file 1 **Nascent DRG neurons arise by differentiation.** Time-lapse confocal projections showing sensory neuron differentiation in a *TgBAC(neurod:EGFP)* embryo between 44 and 52 hpf. The embryo was imaged every six minutes for eight hours. *TgBAC(neurod:EGFP)* is expressed in newly formed DRG neurons. At time zero, there are one to two GFP + cells per ganglion. During the course of imaging, GFP + cells are added to the ganglia by upregulation of the transgene. No GFP + cells are seen to divide during imaging. Scale bar, 20 μm. Click here for file

Additional file 2 **Migrating neural crest cells form the DRG anlage.** Time-lapse confocal projections of a wild-type embryo expressing *Tg(sox10:nlsEos)* transgene in migrating neural crest cells. The embryo was imaged every 10 minutes for 23 hours beginning at 18 hpf. During this time, a subset neural crest cells migrate into the region of the forming DRG and remain there. No neural crest cells migrated into this region after 10 hours of imaging at approximately 28 hpf. Scale bar, 25 μm. Click here for file

Additional file 3 **Neural crest cells divide and differentiate as neurons in the DRG.** Time-lapse confocal projections of the forming DRG in a *Tg(sox10:nlsEos)*/*Tg(neurod:TagRFP)* transgenic embryo between 30 and 56.5 hpf. The embryo was imaged every 10 minutes for 26:30 hours. One cell upregulates *Tg(neurod:TagRFP)* as it differentiates as a neuron. Area boxed at 26:30 is shown again as a 4D projection in close-up, then in reverse with the neuronal precursor tracked from the progenitor cell division. Click here for file

Additional file 4 **Proliferating cells are associated with late larval DRG.** Confocal 1.5 μm slices through an individual ganglion in a 25 dpf, 6.25 mm larval fish. Cells that have incorporated BrdU (red) or are labeled with anti-pH3 antibody (blue) intermingle with Elavl labeled neurons (green). Neurons are not co-labeled with markers of proliferation. Scale bar, 20 μm. Click here for file
